# Fault Diagnosis for Current Sensors in Charging Modules Based on an Adaptive Sliding Mode Observer

**DOI:** 10.3390/s25051413

**Published:** 2025-02-26

**Authors:** Pengfei Huang, Jie Liu, Jiaxin Wang

**Affiliations:** 1 China Three Gorges Corporation Hubei Province, Wuhan 430000, China; huang_pengfei1@ctg.com.cn; 2 Electrical Engineering and Automation, Hefei University of Technology, Hefei 230009, China; 2022170435@mail.hfut.edu.cn

**Keywords:** the charging module of DC charging piles, current sensor, fault diagnosis, adaptive sliding mode observer, adaptive threshold

## Abstract

This article proposes a fault diagnosis method based on an adaptive sliding mode observer (SMO) for current sensors (CSs) in the charging modules of DC charging piles. Firstly, we establish a model of the phase-shift full-bridge (PSFB) converter with CS faults. Secondly, the fault of the CS is reconstructed through system augmentation and non-singular coordinate transformation. Then, an adaptive SMO is designed to estimate the reconstructed state, and the residual between the actual value of the reconstructed state and the observed value is used as the fault detection variable. Finally, by using norms to design adaptive thresholds and comparing them with fault detection variables, the diagnosis of incipient faults, significant faults, and failure faults in CSs can be achieved. The experimental results verify the effectiveness of the proposed method in this paper; the robustness of the method has been verified under the conditions of DC voltage fluctuations and load fluctuations.

## 1. Introduction

The charging module of a DC charging pile, as a crucial piece of equipment for electric vehicle (EV) charging, holds paramount importance in the widespread adoption of EVs (in terms of its stability and reliability) [1,2,3]. Nevertheless, due to the intricate and ever-changing operational environments in which these charging modules reside, their failure rates remain unacceptably high, posing a formidable challenge to the development of EVs. Notably, the CS, an indispensable feedback component within the charging module, poses an immediate threat to the stability of the entire charging system in the event of a malfunction. Consequently, a thorough exploration and investigation into fault diagnosis methods for CSs in charging modules is profoundly significant in ensuring the safe operation of DC charging piles and driving the healthy development of the EV industry. At present, significant achievements have been made in the research of fault diagnosis methods for DC charging piles [4,5].

Currently, fault diagnosis methods for CSs are primarily divided into two categories: data-driven approaches [6,7] and model-based approaches [8,9]. Data-driven fault diagnosis relies heavily on signal processing and artificial intelligence technologies to conduct deep mining and analysis of sensor data, thereby enabling fault detection. For example, reference [10] constructed fault detection variables by extracting fault information from current, voltage, and speed sensors in high-speed trains using principal component analysis, enabling multi-sensor fault diagnosis in high-speed trains. In addition, fault diagnosis and adaptive multichannel fusion calibration of filament CSs for mass spectrometers, based on a convolutional neural network–long short-term memory (CNN-LSTM), is proposed in [11]. Benefiting from the rapid advancements in data processing technologies, methods such as wavelet transform [12], support vector machines [13], and Hilbert transform [14] have been utilized in fault diagnosis. These data-driven methods exhibit flexibility and adaptability, capable of handling complex nonlinear systems and uncertain fault patterns. However, they typically necessitate significant amounts of training data and computational resources and may require retraining and model adjustment for different application scenarios.

Model-based fault diagnosis methods focus on detecting and analyzing sensor faults by establishing mathematical models and observers for the system, typically offering faster diagnosis speeds and lower data requirements. For instance, reference [15] addresses the early fault diagnosis problem for Lipschitz nonlinear systems with sensor biases by decomposing the original system into two subsystems, isolating sensor faults from system disturbances. Subsequently, a total measurable fault information residual is constructed to detect early faults in multiple sensors. Reference [16] initially explored the differences in the mechanisms underlying CS faults and phase break faults, and subsequently introduced a fault diagnosis algorithm that leveraged a neutral point voltage observer. Reference [17] constructed three fault detection variables based on the average normalized value of the product of phase currents in a permanent magnet synchronous motor (PMSM) to achieve fault diagnosis of CSs in the PMSM drive system. Meanwhile, reference [18] first established a proportional full-state observer and then normalized the estimated error calculated from the observed current and actual current to identify changes in the output current rate of the CS in PMSM, thereby determining the fault type. Additionally, methods such as reduced-order observers [19] and fault estimation [20] have been established to achieve typical sensor fault diagnosis. However, existing methods primarily target significant faults in CSs. Due to their low amplitudes and the ease with which they can be confused with various noises, minor faults in CSs are not effectively diagnosed by these methods designed for significant faults. Therefore, research on the diagnosis of minor faults in CSs is highly essential.

Existing research on incipient fault diagnosis methods for various sensors has been widely applied. For instance, in reference [21], a novel interval SMO was constructed by introducing a new reaching law, and an instantaneous fault detection system with a novel residual algorithm and fault detection threshold was proposed. The effectiveness and practicality of this method have been verified through an example involving rectifier DC voltage sensor faults in high-speed railway traction equipment. While the above fault diagnosis methods focus on individual power electronic devices, the charging modules of DC charging piles also necessitate consideration of the impact of high-frequency transformers on inverter and rectifier circuits, leading to significant differences in diagnostic targets compared to the mentioned methods. Consequently, these methods are difficult to apply directly.

It is noteworthy that this paper introduces an innovative fault diagnosis method for CSs in the charging modules of DC charging piles, utilizing an adaptive SMO. The main contributions of this work are as follows:

(1) Fault diagnosis method for CSs: This paper proposes a fault diagnosis method for CSs in the charging modules of DC charging piles based on an adaptive SMO. The proposed method is capable of diagnosing incipient, significant, and failure faults of CSs.

(2) Design of adaptive SMO: By reconstructing CS faults, this paper innovatively introduces a novel adaptive reaching law to design the adaptive SMO. This observer can estimate the reconstructed states and achieve rapid convergence while effectively suppressing high-frequency chattering. Additionally, the residual between the actual and observed values of the reconstructed states is utilized as the fault detection variable, enhancing the accuracy of fault detection.

(3) Adaptive threshold fault diagnosis strategy: To meet the practical requirements under different operating conditions, this paper designs three adaptive thresholds using norms, enabling precise diagnosis of CS faults. Experimental results validate the effectiveness and robustness of this strategy, providing strong support for the safe operation of DC charging piles.

## 2. Modeling

### 2.1. The Charging Module PSFB Converter Model

**Figure 1:**
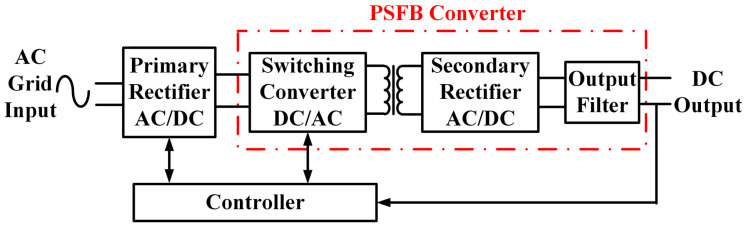
The topology of the charging module.

Figure 1 shows the two-stage conversion electrical structure of the charging module in a DC charging pile. The rectifier circuit, as the front-stage structure, is responsible for rectification and active power factor correction. The PSFB converter is the rear-stage structure, which is responsible for realizing voltage conversion and electrical isolation. Since the PSFB converter achieves high-efficiency energy conversion in the charging module and directly provides DC input to the load, it is an important component of the charging module. Therefore, this paper mainly studies the CS fault diagnosis method of the PSFB converter.

**Figure 2:**
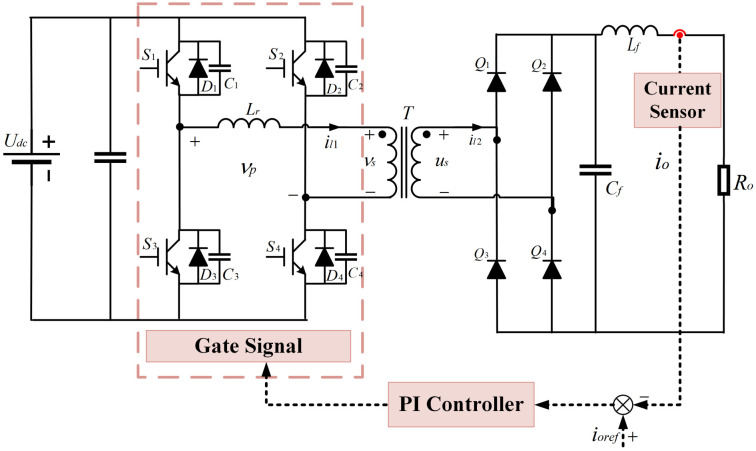
The topology of the PSFB converter.

The PSFB converter, as illustrated in Figure 2, is composed of an inverter bridge, a high-frequency transformer *T*, a rectifier bridge, and a filter [22]. The inverter side of the PSFB converter utilizes a fully controlled full-bridge inverter circuit, where the power modules within the inverter bridge each contain four switching tubes, $S_{1},S_{2},S_{3},S_{4}$, parasitic capacitors, $C_{1},C_{2},C_{3},C_{4}$, and anti-parallel diodes, $D_{1},D_{2},D_{3},D_{4}$. Each switching tube is paralleled with a parasitic capacitor and an anti-parallel diode. Between the midpoints of the bridge arms, a series resonant inductor and the primary winding of the high-frequency transformer are connected. The rectifier side employs a non-controllable full-bridge rectifier circuit, with the rectifier bridge consisting of four diodes, $Q_{1},Q_{2},Q_{3},Q_{4}$. The secondary winding of the high-frequency transformer serves as the input for the rectifier bridge. The voltage ratio between the primary and secondary sides of the high-frequency transformer is $\nu_{s}\left(t\right)$:$u_{s}\left(t\right)$ = 2.5:1. The filter module, which comprises filter inductors and filter capacitors, $R_{o}$, serves as the load for the PSFB converter.

According to the working principle of the PSFB converter, the mathematical model of the PSFB converter can be expressed as follows [23]:

(1) $$\left\{\begin{array}{ccc} \dot{x} & = & Ax+B_{1}u_{1}+B_{2}u_{2}+B_{3}u_{3}\\ y & = & Cx \end{array}\right.$$

where $x=i_{o}$, $u_{1}=\nu_{p}$, $u_{2}=i_{l1}$, u3=dil1u3=dil1dtdt, $A=-R_{o}/L$, B1=112.5Lsgnil22.5Lsgnil2, $\mathrm{sgn}\left(i_{l2}\right)$ is the signum function. $i_{l2}$ is the current on the secondary side of the high-frequency transformer *T*. *L* is the equivalent inductance value of the leakage inductance of the transformer and the resonant inductance of the circuit. B2=RtRt2.5Lsgnil22.5Lsgnil2, $R_{t}$ is the equivalent resistance value of the resonant components as well as the parasitic resistance of the transformer. B3=112.5sgnil22.5sgnil2, C=1.

### 2.2. Fault Description

While the PSFB converter is in operation, the aging and degradation of its internal components are inevitable phenomena that occur over time. Among these components, the classification of current CS failures depends on the comparison between the magnitude of the fault current with the magnitude of the normal output current. This classification is divided into the following three separate categories: incipient fault stage, significant fault stage, and failure stage. To simulate the occurrence of CS faults, we represent CS fault evolution model as follows [24]:

(2) $$F_{s}=\int_{0}^{t}e^{A_{s}\left(t-\tau \right)}\phi d\tau$$

In this model, the variable $F_{s}$ denotes the malfunction within the CS, and ϕ stands for the excitation signal of the fault, influencing its magnitude. Moreover, $A_{s}$ represents the coefficient for fault amplitude, dictating the rate at which the magnitude of faults varies. According to (2), the CS fault $F_{s}$, with the specific form, is given as follows:

(3) $${\dot{F}}_{s}=A_{s}F_{s}+\phi$$

Additionally, the following fault amplitude variable Γ is defined to represent the degree of fault in the CS:

(4) $$\Gamma =\frac{∥F_{s}∥}{∥i_{o}∥}\times 100\%$$

In the equation, $∥F_{s}∥$ represents the fault amplitude, while $∥i_{o}∥$ denotes the amplitude of the output current, which is generally required to be within the rated load range with the THD of the output current lower than 5%.

- When 5%≤Γ≤15%, the sensor is defined as being in a state of incipient fault. At this stage, the output value deviates slightly from the normal value, indicating the initial stage of the fault. Due to the small fault amplitude, the impact on the system is relatively minor.
- When 15%≤Γ≤50%, the sensor is defined as being in a state of significant fault. At this stage, the output value deviates significantly from the normal value, indicating the intermediate stage of the fault. Due to the larger fault amplitude, it can significantly affect the stability of the system and reduce its performance.
- When Γ>50%, the sensor is defined as being in a state of failure. At this stage, the output value deviates greatly from the normal value, indicating the final stage of the fault. Due to the very large fault amplitude, it can severely impact the stability of the system and potentially lead to system collapse.

Combining (1) and (3), while also considering the presence of unknown disturbances such as external measurement noise in the actual operation of the PSFB converter, the mathematical model of the PSFB converter with a faulty CS can be expressed as follows:

(5) $$\left\{\begin{array}{ccc} \dot{x} & = & Ax+B_{1}u_{1}+B_{2}u_{2}+B_{3}u_{3}+Gd\\ y & = & Cx+KF_{s} \end{array}\right.$$

In the equation, $F_{s}$ represents the fault in the output CS of the PSFB converter, *K* is the coefficient matrix of the fault $F_{s}$, and *G* is the coefficient matrix of the disturbances. It is important to note that CSs may be prone to unknown disturbances and system noise during the actual measurement process. To accurately represent the measured values, d(t) is introduced.

## 3. CS Fault Diagnosis Methods

**Figure 3:**
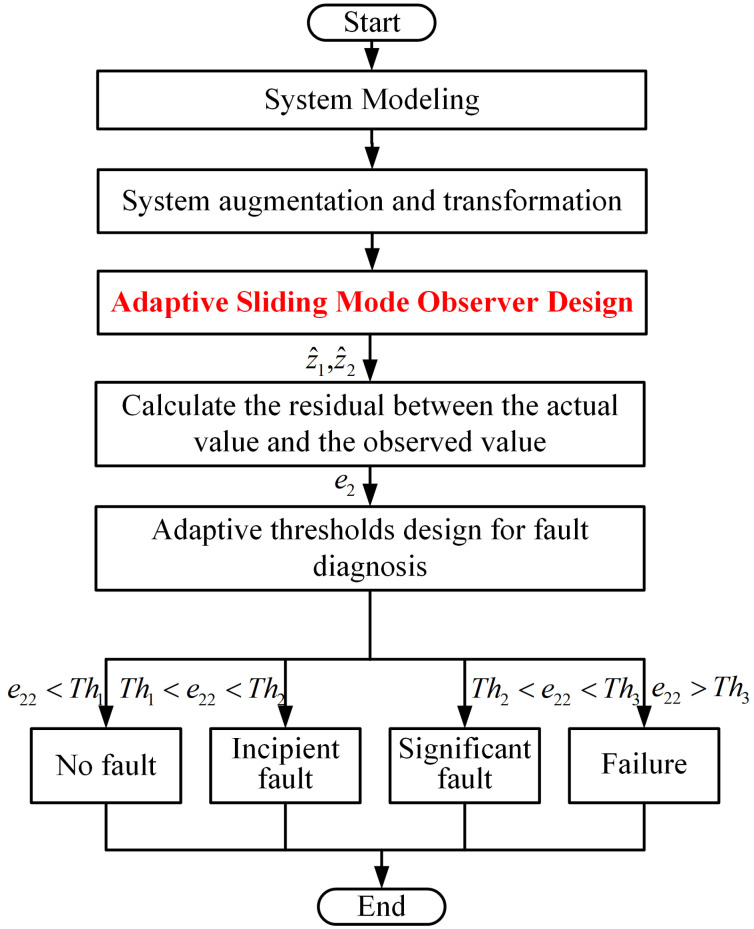
Schematic diagram of the fault diagnosis.

Based on the mathematical model proposed in (5), this paper presents a fault diagnosis method for CSs in the charging modules of DC charging piles using an adaptive SMO. The diagnostic principle is illustrated in Figure 3. Firstly, the mathematical model of the PSFB converter circuit within the charging module, which contains a faulty CS, undergoes state augmentation to obtain an augmented system. This augmentation enables the system to more comprehensively reflect the state information of the CS. Secondly, a non-singular coordinate transformation is applied to the augmented system to reconstruct the CS fault, making the fault characteristics more prominent and facilitating subsequent observation and diagnosis. Then, the adaptive SMO is established to estimate the reconstructed state. By incorporating an innovative adaptive reaching law, the observer attains swift convergence and efficiently mitigates high-frequency chattering. The fault detection variable is represented by the difference between the actual and observed values of the reconstructed state. Finally, an adaptive threshold is designed using norm theory to realize the fault diagnosis of the CS.

### 3.1. System Augmentation and Transformation

To achieve fault detection of the CS in the PSFB converter, the mathematical model (5) containing the CS fault is augmented to obtain the following augmented system [25]:

(6) $$\left\{\begin{array}{ccc} \dot{\bar{x}} & = & \bar{A}\bar{x}+{\bar{B}}_{1}u_{1}+{\bar{B}}_{2}u_{2}+{\bar{B}}_{3}u_{3}+\bar{G}d+\bar{E}\phi \\ y & = & \bar{C}x \end{array}\right.$$

where $\bar{A}=diag\left(\begin{array}{cc} A & A_{s} \end{array}\right)$, $\bar{x}=\left(\begin{array}{c} x\\ F_{s} \end{array}\right)$, ${\bar{B}}_{1}=\left(\begin{array}{c} B_{1}\\ 0 \end{array}\right)$, ${\bar{B}}_{2}=\left(\begin{array}{c} B_{2}\\ 0 \end{array}\right)$, ${\bar{B}}_{3}=\left(\begin{array}{c} B_{3}\\ 0 \end{array}\right)$, $\bar{C}=\left(\begin{array}{cc} C & K \end{array}\right)$, $\bar{G}=\left(\begin{array}{c} G\\ 0 \end{array}\right)$, $\bar{E}=\left(\begin{array}{c} 0\\ I \end{array}\right)$.

**Lemma** **1** ([26]). *There exists a non-singular transformation matrix, P, such that $P\bar{A}P^{-1}=\left(\begin{array}{cc} A_{1} & A_{2}\\ A_{3} & A_{4} \end{array}\right)$, $P{\bar{B}}_{1}=\left(\begin{array}{c} B_{11}\\ B_{12} \end{array}\right)$, $P{\bar{B}}_{1}=\left(\begin{array}{c} B_{11}\\ B_{12} \end{array}\right)$, $P{\bar{B}}_{2}=\left(\begin{array}{c} B_{21}\\ B_{22} \end{array}\right)$, $P{\bar{B}}_{3}=\left(\begin{array}{c} B_{31}\\ B_{32} \end{array}\right)$, $P\bar{G}=\left(\begin{array}{c} G_{1}\\ G_{2} \end{array}\right)$, $P\bar{E}=\left(\begin{array}{c} 0\\ E_{2} \end{array}\right)$.*

*According to Lemma 1, if a non-singular coordinate transformation $z=P\bar{x}={\left(\begin{array}{cc} z_{1} & z_{2} \end{array}\right)}^{T}$ is performed on the augmented matrix, the result of the system (6) after the coordinate transformation is as follows:*

(7) $$\left\{\begin{array}{cc} {\dot{z}}_{1}= & A_{1}z_{1}+A_{2}z_{2}+B_{11}u_{1}+B_{21}u_{2}+B_{31}u_{3}+G_{1}d\\ {\dot{z}}_{2}= & A_{3}z_{1}+A_{4}z_{2}+B_{12}u_{1}+B_{22}u_{2}+B_{32}u_{3}+G_{2}d+E_{2}\phi \\ y= & C_{1}z_{1}+C_{2}z_{2} \end{array}\right.$$

### 3.2. Adaptive SMO Design

Based on the non-singularly transformed system (7), we design an adaptive SMO:

(8) $$\left\{\begin{array}{cc} {\dot{\hat{z}}}_{1}= & A_{1}{\hat{z}}_{1}+A_{2}{\hat{z}}_{2}+B_{11}u_{1}+B_{21}u_{2}+B_{31}u_{3}+K_{1}\left(z_{1}-{\hat{z}}_{1}\right)+v\left(s_{1}\right)\\ {\dot{\hat{z}}}_{2}= & A_{3}{\hat{z}}_{1}+A_{4}{\hat{z}}_{2}+B_{12}u_{1}+B_{23}u_{2}+B_{32}u_{3}+K_{2}\left(z_{2}-{\hat{z}}_{2}\right)+v\left(s_{2}\right)\\ \hat{y}= & C_{1}{\hat{z}}_{1}+C_{2}{\hat{z}}_{2} \end{array}\right.$$

In the expressions, ${\hat{z}}_{1},{\hat{z}}_{2}$ are the observed values of $z_{1},z_{2}$, while $K_{1},K_{2}$ are the parameters to be designed, satisfying $A_{1}-K_{1}<0$ and $A_{4}-K_{2}<0$. $v\left(s_{1}\right),v\left(s_{2}\right)$ represent the designed adaptive reaching laws. To enhance the approaching speed and diminish high-frequency chattering, the adaptive reaching laws $v\left(s_{1}\right),v\left(s_{2}\right)$ are formulated as outlined below:

(9) $$v\left(s_{1}\right)=k_{1}/\left(\lambda +\left(1+{\left|s_{1}\right|}^{-1}-\lambda \right)e^{-\alpha {\left|S_{1}\right|}^{\,}}\right)\tanh\left(s_{1}\right)$$

(10) $$v\left(s_{2}\right)=k_{2}/\left(\lambda +\left(1+{\left|s_{2}\right|}^{-1}-\lambda \right)e^{-\alpha {\left|S_{2}\right|}^{\,}}\right)\tanh\left(s_{2}\right)$$

where $k_{1},k_{2}$ are the designed sliding mode gains, λ,α are positive constants, $\tanh\left(s\right)=\frac{e^{s}-e^{-s}}{e^{s}+e^{-s}}$, with the condition that 0<λ<1, and $s_{1},s_{2}$ represent the sliding surfaces to be determined.

### 3.3. Calculate the Residual

Based on this, taking the residual $e_{1}=z_{1}-{\hat{z}}_{1}$ and $e_{2}=z_{2}-{\hat{z}}_{2}$, we can obtain the following error dynamic system from (7) and (8):

(11) $$\left\{\begin{array}{cc} {\dot{e}}_{1}= & \left(A_{1}-K_{1}\right)e_{1}+A_{2}e_{2}+G_{1}d-v\left(s_{1}\right)\\ {\dot{e}}_{2}= & A_{3}^{\,}e_{1}+\left(A_{4}-K_{2}\right)e_{2}+G_{2}^{\,}d+E_{2}\phi -v\left(s_{2}\right)\\ \hat{y}= & C_{1}{\hat{z}}_{1}+C_{2}{\hat{z}}_{2} \end{array}\right.$$

According to (11), the expression in the error dynamic system ${\dot{e}}_{2}$ contains the fault excitation signal ϕ, meaning that ${\dot{e}}_{2}$ can directly reflect the magnitude of the CS fault. The residual $e_{2}$ can be selected as the fault detection variable, and an adaptive threshold for CS fault diagnosis can be designed based on $e_{2}$.

To prove the asymptotic stability of the error dynamic system shown in (11), Theorem 1 is presented.

**Theorem** **1.** *When the sensor fault does not occur, if there exist $P_{1},P_{2}$, and $K_{1},K_{2}$ that satisfy the following conditions:*

$$\begin{array}{c} P_{1}\left(A_{1}-K_{1}\right)+{\left(A_{1}-K_{1}\right)}^{T}P_{1}<0\\ P_{2}\left(A_{4}^{\,}-K_{2}\right)+{\left(A_{4}^{\,}-K_{2}\right)}^{T}P_{2}<0\\ P_{1}A_{2}+A{\,}_{3}^{T}P_{2}<0 \end{array}$$

*Then the error dynamic system is asymptotically stable, and the adaptive SMO can estimate the system output current.*

**Proof** **of** **Theorem** **1.** Taking the Lyapunov function $V_{1}=e_{1}^{T}P_{1}e_{1}$ and $V_{2}=e_{2}^{T}P_{2}e_{2}$, with the conditions $∥\nu \left(s_{1}\right)∥>∥G_{1}d∥$ and $∥\nu \left(s_{2}\right)∥>∥G_{2}d∥$, from the error dynamic system (11), we can derive the following:

(12) $$\begin{array}{cc} {\dot{V}}_{1}= & {\dot{e}}_{1}^{T}P_{1}e_{1}+e_{1}^{T}P_{1}{\dot{e}}_{1}\\ = & \left(\left(A_{1}-K_{1}\right)e_{1}+A_{2}e_{2}+G_{1}d\right.{\left.-\nu \left(s_{1}\right)\right)}^{T}P_{1}e_{1}+e_{1}^{T}P_{1}\left(\left(A_{1}-K_{1}\right)e_{1}+A_{2}e_{2}+G_{1}d\right.\left.-\nu \left(s_{1}\right)\right)\\ = & e_{1}^{T}\left(P_{1}\left(A_{1}-K_{1}\right)+{\left(A_{1}-K_{1}\right)}^{T}P_{1}\right)e_{1}+2e_{1}^{T}P_{1}A_{2}e_{2}+2e_{1}^{T}P_{1}\left(G_{1}d-\nu \left(s_{1}\right)\right)\\ \leq & e_{1}^{T}\left(P_{1}\left(A_{1}-K_{1}\right)+{\left(A_{1}-K_{1}\right)}^{T}P_{1}\right)e_{1}+2e_{1}^{T}P_{1}A_{2}e_{2} \end{array}$$

(13) $$\begin{array}{cc} {\dot{V}}_{2}= & {\dot{e}}_{2}^{T}P_{2}e_{2}+e_{2}^{T}P_{2}{\dot{e}}_{2}\\ = & \left(A_{3}^{\,}e_{1}+\left(A_{4}-K_{2}\right)e_{2}+G_{2}^{\,}d\right.+E_{2}\varphi {\left.-\nu \left(s_{2}\right)\right)}^{T}P_{2}e_{2}+e_{2}^{T}P_{2}\left(A_{3}^{\,}e_{1}+\left(A_{4}-K_{2}\right)e_{2}+G_{2}^{\,}d\right.+E_{2}\varphi \left.-\nu \left(s_{2}\right)\right)\\ = & e_{2}^{T}\left(P_{2}\left(A_{4}-K_{2}\right)+{\left(A_{4}-K_{2}\right)}^{T}P_{2}\right)e_{2}+2e_{2}^{T}P_{2}A_{3}^{\,}e_{1}+2e_{2}^{T}P_{2}\left(G_{2}d+E_{2}\varphi -\nu \left(s_{2}\right)\right)\\ \leq & e_{2}^{T}\left(P_{2}\left(A_{4}-K_{2}\right)+{\left(A_{4}-K_{2}\right)}^{T}P_{2}\right)e_{2}+2e_{2}^{T}P_{2}A_{3}^{\,}e_{1} \end{array}$$

(14) $$\begin{array}{cc} \dot{V}= & {\dot{V}}_{1}+{\dot{V}}_{2}\\ = & \left(\left(A_{1}-K_{1}\right)e_{1}+A_{2}^{\,}e_{2}+G_{1}^{\,}d\right.{\left.-\nu \left(s_{1}\right)\right)}^{T}P_{1}e_{1}+e_{1}^{T}P_{1}\left(\left(A_{1}-K_{1}\right)e_{1}+A_{2}e_{2}+G_{1}d\right.\left.-\nu \left(s_{1}\right)\right)\\ \; & +\left(A_{3}^{\,}e_{1}+\left(A_{4}-K_{2}\right)e_{2}+G_{2}^{\,}d\right.+E_{2}\varphi {\left.-\nu \left(s_{2}\right)\right)}^{T}P_{2}e_{2}+e_{2}^{T}P_{2}\left(A_{3}^{\,}e_{1}+\left(A_{4}-K_{2}\right)e_{2}+G_{2}^{\,}d\right.+E_{2}\varphi \left.-\nu \left(s_{2}\right)\right)\\ \leq & e_{1}^{T}\left(P_{1}\left(A_{1}-K_{1}\right)+{\left(A_{1}-K_{1}\right)}^{T}P_{1}\right)e_{1}+e_{2}^{T}\left(P_{2}\left(A_{4}-K_{2}\right)+{\left(A_{4}-K_{2}\right)}^{T}P_{2}\right)e_{2}+2e_{1}^{T}\left(P_{1}A_{2}^{\,}+A{{\,}_{3}^{\,}}^{T}P_{2}\right)e_{2}\\ = & e^{T}\Pi e \end{array}$$

where $\Psi_{1}=P_{1}\left(A_{1}-K_{1}\right)+{\left(A_{1}-K_{1}\right)}^{T}P_{1}$, $\Psi_{2}=P_{1}A_{2}+A{\,}_{3}^{T}P_{2}$, $\Psi_{3}=P_{2}\left(A_{4}^{\,}-K_{2}\right)+{\left(A_{4}^{\,}-K_{2}\right)}^{T}P_{2}$, $\Pi =\left(\begin{array}{cc} \Psi_{1} & \Psi_{2}\\ \Psi_{2}^{T} & \Psi_{3} \end{array}\right)$, $e={\left(\begin{array}{cc} e_{1} & e_{2} \end{array}\right)}^{T}$ Since the conditions $A_{1}-K_{1}<0$ and $A_{4}-K_{2}<0$ are met when constructing the adaptive SMO, it can be concluded from the above that $\dot{V}<0$, which means that the error of the error dynamic system (11) will eventually converge to 0, indicating that (11) is asymptotically stable. The proof is complete. □

### 3.4. Adaptive Threshold Design for Fault Diagnosis

Based on the adaptive SMO designed above, $∥e_{2}∥$ incorporating the fault excitation signal ϕ is utilized to construct the fault detection variable. According to (11), $e_{2}$ can be calculated, and the expression of $e_{2}$ is given as follows:

(15) $$\begin{array}{cc} e_{2}= & \int_{0}^{t}e^{(A_{4}^{\,}-K_{2})t}\left(A_{3}^{\,}e_{1}+G_{2}^{\,}d+E_{2}\phi -v\left(s_{2}\right)\right)dr+e^{(A_{4}^{\,}-K_{2})t}e_{2}\left(0\right) \end{array}$$

Utilizing the properties of the norm triangle inequality, the following can be derived from (18):

(16) $$\begin{array}{cc} ∥e_{2}∥\leq & \int_{0}^{t}e^{\lambda_{s}t}\left(∥A_{3}^{\,}∥∥e_{1}∥+∥G_{2}^{\,}d∥+∥E_{2}∥∥\phi ∥\right)dr+e^{\lambda_{s}t}\omega_{s} \end{array}$$

where $\lambda_{s}$ satisfies $e^{(A_{4}^{\,}-K_{2})t}<e^{\lambda_{s}t}$, and $\omega_{s}$ is the upper bound of $∥e_{2}\left(0\right)∥$, $∥e_{2}\left(0\right)∥\leq \omega_{s}$.

On this basis, adaptive thresholds for different stages are designed based on the evolution progress of the CS fault in the PSFB converter circuit, aiming to achieve more accurate diagnostic results. It is essential to emphasize that the process of selecting parameters for the adaptive thresholds involves a meticulous balance between the sensitivity and robustness of fault detection, aimed at preventing both false negatives and false positives.

- In the stage of incipient fault in the CS of the PSFB converter circuit, where 5%≤Γ≤15%, the adaptive threshold, $Th_{1}$, for detecting incipient faults is designed as follows, based on (19):
  (17) $$\begin{array}{cc} Th_{1} & =\int_{0}^{t}e^{\lambda_{s}t}\left(∥A_{3}^{\,}∥∥e_{1}∥+∥G_{2}^{\,}d∥+∥E_{2}∥\phi_{1}\right)dr+e^{\lambda_{s}t}\omega_{s} \end{array}$$
  where $\phi_{1}$ represents the critical value of the fault excitation signal ϕ corresponding to the occurrence of an incipient fault in the CS. Assuming $T_{1}$ is the time when the incipient fault in CS occurs, the diagnosis time $T_{th1}$ for this incipient fault in the sensor can be expressed as follows:
  (18) $$\begin{array}{c} T_{th1}=\inf\left\{t>T_{1}|\left|e_{2}\right|>Th_{1}\right\} \end{array}$$
- In the stage of significant fault in the CS of the PSFB converter circuit, where 15%≤Γ≤50%, the adaptive threshold $Th_{2}$ for detecting significant faults is designed as follows, based on (19):
  (19) $$\begin{array}{c} Th_{2}=\int_{0}^{t}e^{\lambda_{s}t}\left(∥A_{3}^{\,}∥∥e_{1}∥+∥G_{2}^{\,}d∥+∥E_{2}∥\phi_{2}\right)dr+e^{\lambda st}\omega_{s} \end{array}$$
  where $\phi_{2}$ represents the critical value of the fault excitation signal ϕ corresponding to the occurrence of a significant fault in the CS. Assuming $T_{2}$ is the time when the significant fault in the sensor occurs, the diagnosis time $T_{th2}$ for this significant fault in the sensor can be expressed as follows:
  (20) $$\begin{array}{c} T_{th2}=\inf\left\{t>T_{2}|\left|e_{2}\right|>Th_{2}\right\} \end{array}$$
- In the failure state of the CS in the PSFB converter circuit, where Γ>50%, the adaptive threshold $Th_{3}$ for detecting the failure fault is designed as follows, based on Equation (19):
  (21) $$\begin{array}{c} Th_{3}=\int_{0}^{t}e^{\lambda_{s}t}\left(∥A_{3}^{\,}∥∥e_{1}∥+∥G_{2}^{\,}d∥+∥E_{2}∥\phi_{3}\right)dr+e^{\lambda_{s}t}\omega_{s} \end{array}$$
  where $\phi_{3}$ represents the critical value of the fault excitation signal ϕ corresponding to the occurrence of the CS failure. Given that $T_{3}$ is the time when the sensor failure occurs, the diagnosis time $T_{th3}$ for the sensor failure fault can be calculated as follows:
  (22) $$\begin{array}{c} T_{th3}=\inf\left\{t>T_{3}|\left|e_{2}\right|>Th_{3}\right\} \end{array}$$

## 4. Experimental Verification

**Figure 4:**
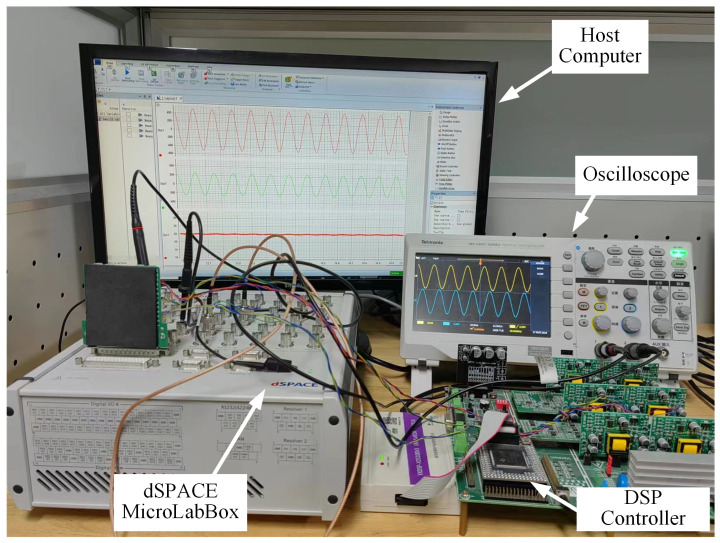
HIL experimental device.

To validate the effectiveness and robustness of the fault diagnosis method proposed in this paper, the HIL simulation experimental setup, as illustrated in Figure 4, was employed for experimental verification. This experimental setup comprises a dSPACE MicroLabBox, a host computer, a digital signal processor (DSP) controller with the model TMS320F28335, and an oscilloscope. Table 1 provides the detailed key parameters of the PSFB converter used in the experiments.

### 4.1. Fault Diagnosis of Faults in CSs

#### 4.1.1. Fault Diagnosis of CS Incipient Faults

**Figure 5:**
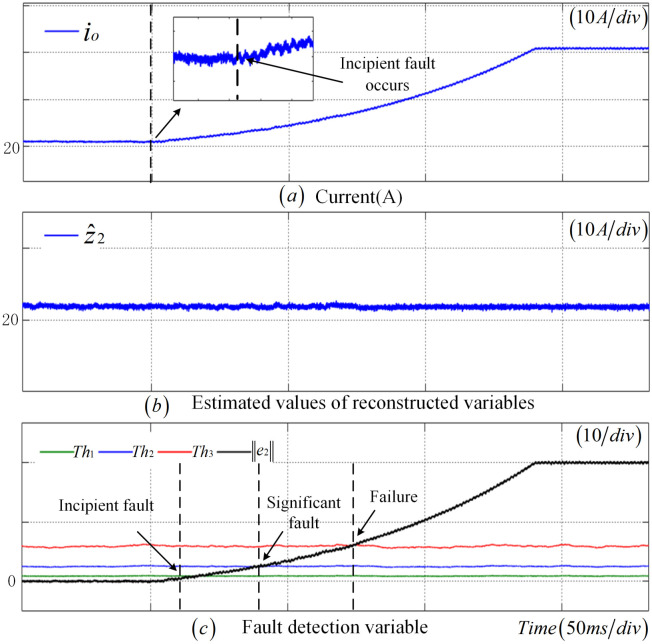
Incipient fault diagnosis results of CS.

This section validates the efficacy of the proposed fault diagnosis methodology by exemplifying an incipient fault in the CS. As evident from Figure 5, subsequent to the occurrence of an incipient fault in the CS at t=0.265 s, $z_{2}$ gradually increases with the augmentation of the fault amplitude, while ${\hat{z}}_{2}$ remains largely stable, resulting in a continuous escalation of the $∥e_{2}∥$. At t=0.3 s, $∥e_{2}∥$ surpasses $Th_{1}$, suggesting the presence of an incipient fault in the CS. As the fault amplitude continues to intensify, $∥e_{2}∥$ exceeds $Th_{2}$ at 0.35 s, signifying a significant fault in the CS. Finally, at t=0.438 s, $∥e_{2}∥$ once more transcends the adaptive threshold $Th_{3}$, indicating that the CS is in a state of failure.

#### 4.1.2. Fault Diagnosis of CS Offset Faults

**Figure 6:**
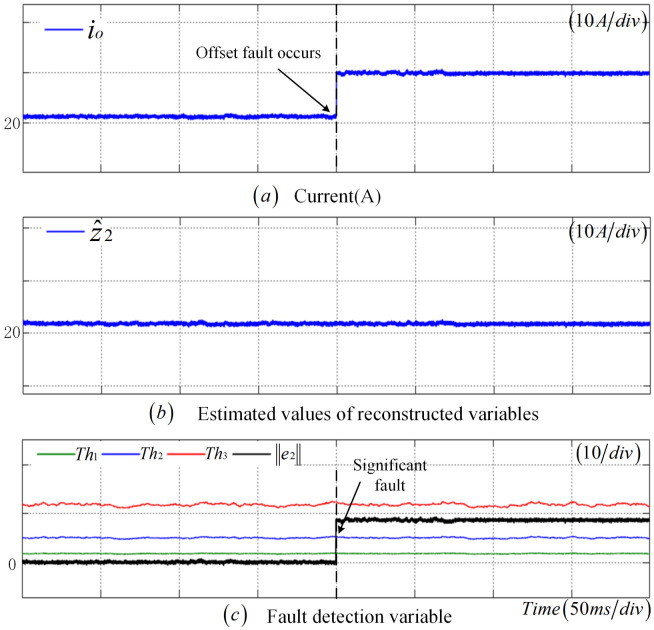
Offset fault diagnosis results of CS.

As shown in Figure 6, at t=0.25 s, a significant deviation between the measurement result of the output CS and the actual value is observed. Moreover, $z_{2}$ varies with the change in fault amplitude, while ${\hat{z}}_{2}$ remains largely unchanged. Moreover, $∥e_{2}∥$ also exhibits a notable change. At t=0.254 s, $∥e_{2}∥$ surpasses the adaptive threshold $Th_{2}$, with a detection time of 4 ms, confirming that the CS is experiencing a significant fault condition.

#### 4.1.3. Fault Diagnosis of CS Stuck Faults

**Figure 7:**
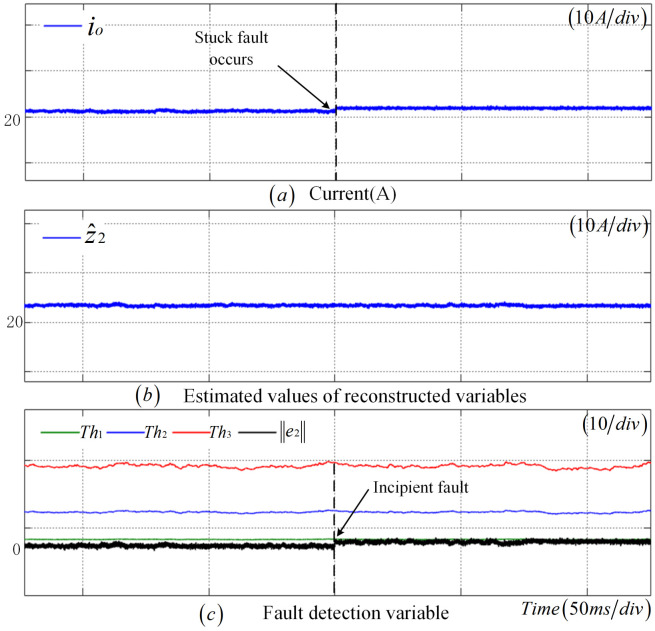
Stuck fault diagnosis results of CS.

As depicted in Figure 7, a stuck fault occurs in the CS at 0.25 s. Due to the subtle fluctuations in the output value of the CS, $z_{2}$ undergoes a transformation according to the fault amplitude, while ${\hat{z}}_{2}$ remains largely unchanged. Notably, $∥e_{2}∥$ exhibits a significant change. At t=0.252 s, $∥e_{2}∥$ surpasses the adaptive threshold, $Th_{1}$, indicating that the CS is in an incipient fault state, with a detection time of 2 ms.

#### 4.1.4. Fault Diagnosis of CS Disconnection Faults

**Figure 8:**
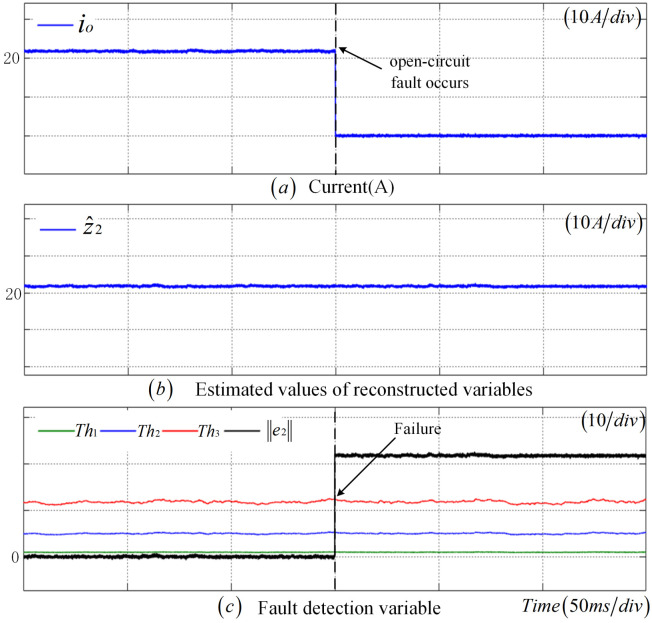
Disconnection fault diagnosis results of CS.

As illustrated in Figure 8, upon the occurrence of a disconnection fault in CS at 0.25 s, the measured value of the CS drops to zero, resulting in a pronounced change in $z_{2}$. Meanwhile, ${\hat{z}}_{2}$ remains largely unchanged, and $∥e_{2}∥$ also exhibits a significant variation. Notably, $∥e_{2}∥$ surpasses the adaptive thresholds $Th_{1}$, $Th_{2}$, and $Th_{3}$ almost simultaneously, with a detection time of 5 ms, indicating a fault in the CS.

### 4.2. Robustness Verification

#### 4.2.1. DC-Side Voltage Fluctuations

**Figure 9:**
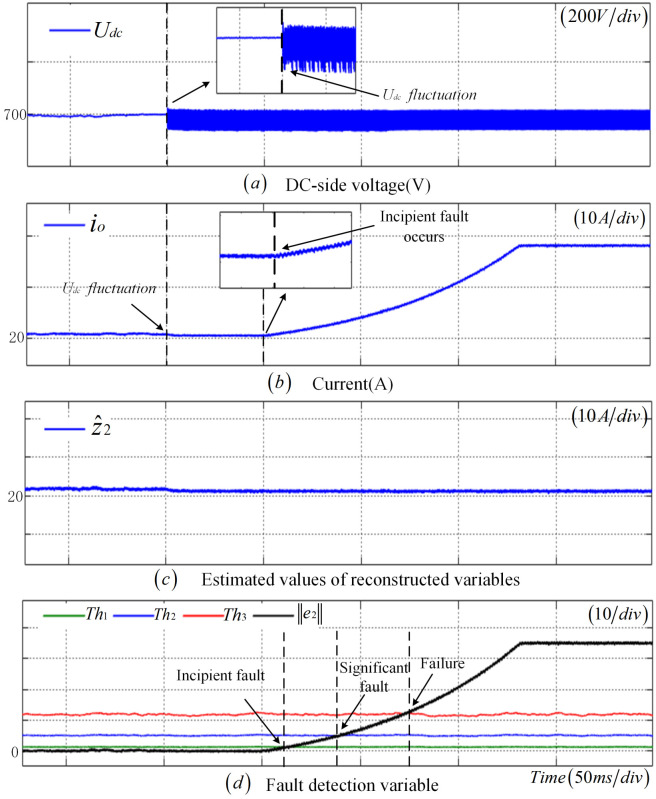
Robustness verification results of the CS incipient fault under DC-side voltage fluctuations.

**Figure 10:**
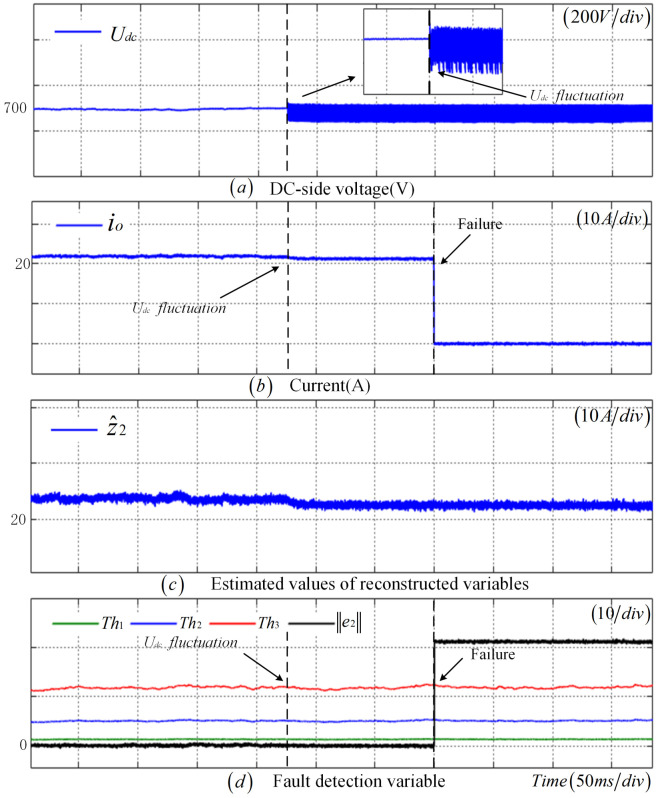
Robustness verification results of the CS disconnection fault under the DC-side voltage fluctuation.

- To verify the robustness of the fault detection method proposed in this paper, Figure 9 presents the diagnosis results of the incipient fault in the CS under DC-side voltage fluctuations. In this scenario, a DC-side voltage fluctuation is introduced at t=1 s, followed by an incipient fault in the CS occurring at t=1.5 s. Upon the introduction of the DC-side voltage fluctuation at t=1 s, the output current experiences slight fluctuations; however, $∥e_{2}∥$ remains close to zero, indicating no false alarm. Subsequently, when the CS fault occurs at 1.5 s, as depicted in Figure 9d, $∥e_{2}∥$ sequentially exceeds $Th_{1}$, $Th_{2}$, and $Th_{3}$, demonstrating that the proposed fault detection method successively identifies the occurrence of an incipient fault, a significant fault, and a complete failure in the sensor.
- To demonstrate the robustness of the fault detection method introduced in this paper, Figure 10 displays the diagnostic results for a CS disconnection fault under $U_{dc}$ fluctuations. In detail, $U_{dc}$ fluctuations are introduced at t=1 s, followed by a CS disconnection fault at t=1.5 s. Despite slight fluctuations in the output current following the $U_{dc}$ fluctuations after 1 s, $∥e_{2}∥$ remains stable near zero and avoids triggering any false alarms. Nevertheless, upon the occurrence of the CS failure at t=1.5 s, Figure 10d clearly illustrates that $∥e_{2}∥$ surpasses the $Th_{3}$ rapidly, confirming the successful detection of the sensor failure by the fault detection method presented in this paper.

#### 4.2.2. Load Fluctuations

**Figure 11:**
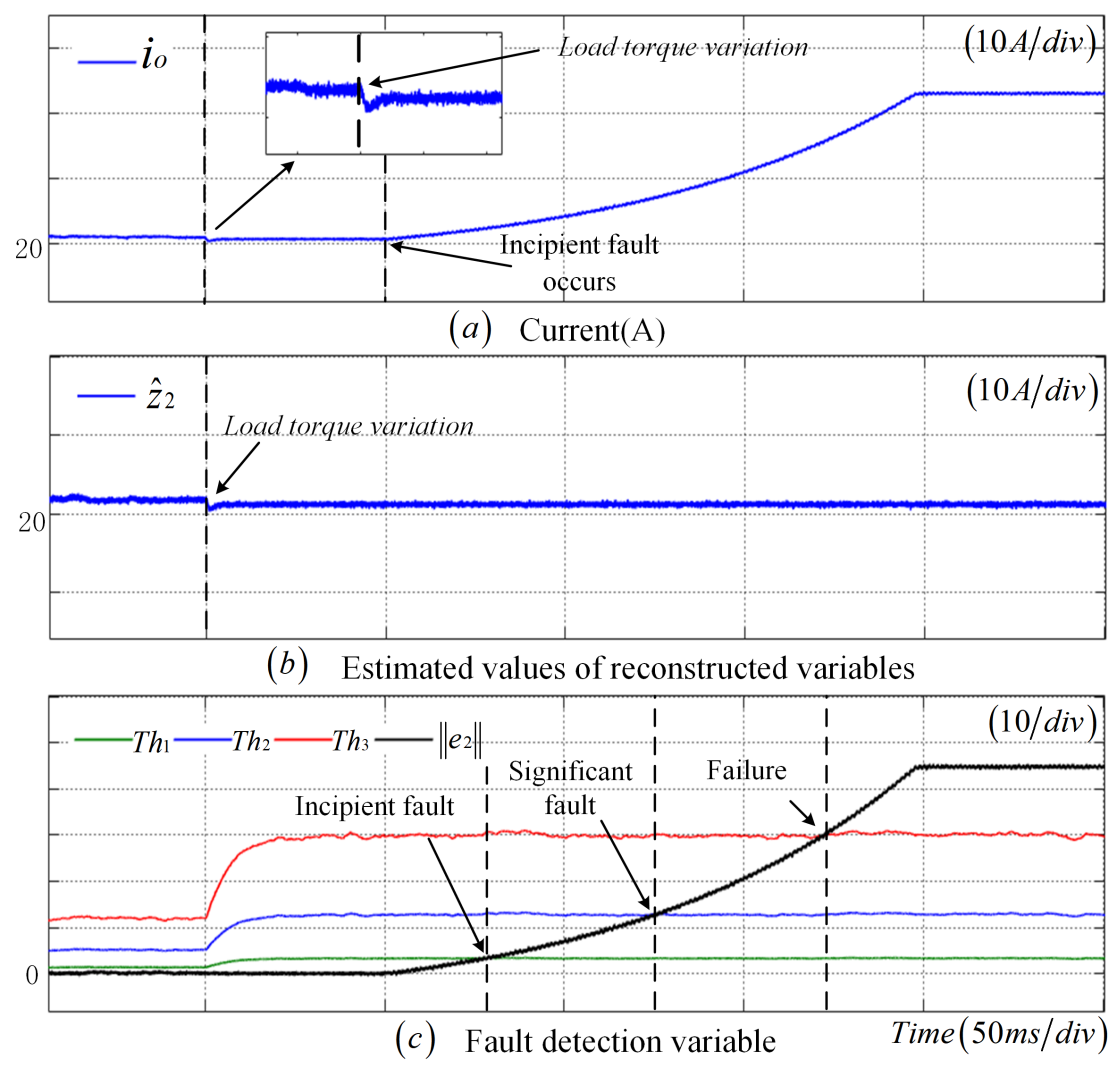
Robustness verification results of the CS incipient fault under load torque variation.

**Figure 12:**
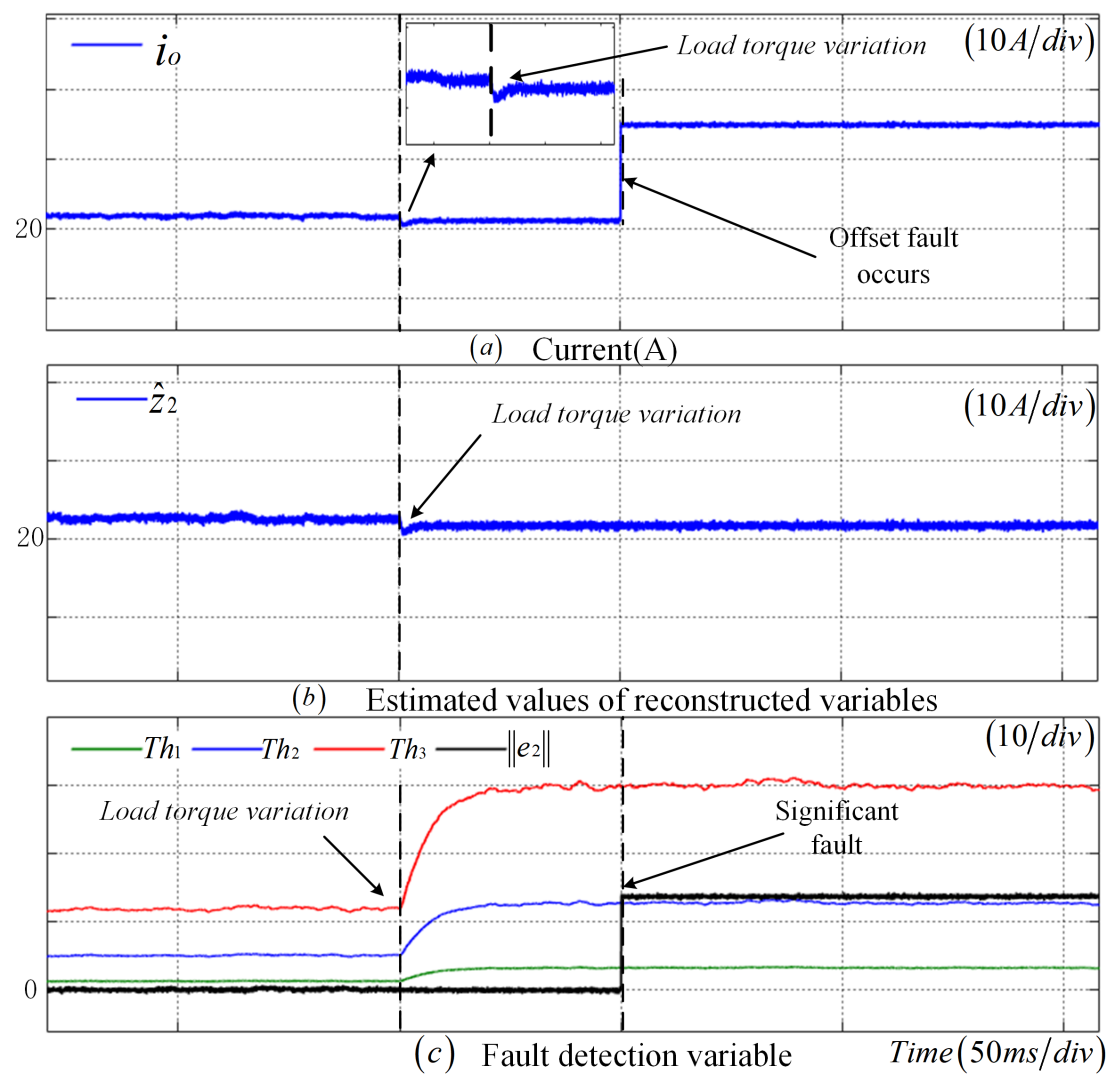
Robustness verification results of the CS offset fault under load torque variation.

- To validate the robustness of the fault detection method proposed in this paper under load fluctuations, Figure 11 presents the diagnostic results of an incipient fault in the CS when the load resistance abruptly changes. Specifically, at t=1 s, the load increases to 1.2 times its original value, and an incipient fault occurs in the CS at 1.4 s. Following the load fluctuation, there is a slight variation in the output current, and the fault detection thresholds undergo significant changes. Nevertheless, $∥e_{2}∥$ does not exceed the threshold, thus avoiding false alarms. When the CS fault occurs at 1.5 s, as depicted in Figure 10 and Figure 11c, the observer residuals sequentially exceed $Th_{1}$, $Th_{2}$, and $Th_{3}$, indicating the detection of an incipient fault, a significant fault, and a complete failure in the sensor, respectively.
- To assess the robustness of the fault detection approach introduced in this paper when subjected to load torque variation, Figure 12 demonstrates the diagnostic results of a CS offset fault scenario where the load torque abruptly rises to 1.2 times its initial value. The load change takes place at t=1 s, followed by the occurrence of a CS offset fault at t=1.5 s. This load torque variation leads to minor fluctuations in the output current and necessitates a substantial adjustment in the fault detection threshold. Nevertheless, $∥e_{2}∥$ remains below the predefined threshold, thereby avoiding any false alarms. Upon the actual manifestation of the CS fault at t=1.5 s, as depicted in Figure 12c, the observer’s residual experiences a rapid increase and exceeds $Th_{2}$, confirming the successful identification of the significant fault by the fault detection method presented in this paper.

The final results of the aforementioned experiments demonstrate that the innovative fault diagnosis method proposed in this paper exhibits exceptional robustness when dealing with complex and variable interference scenarios such as $U_{dc}$ fluctuations and load torque variation. This method does not generate false alarms under adverse conditions and maintains a high level of diagnostic stability across various interference scenarios, ensuring the continuity and reliability of diagnostic results. Moreover, it demonstrates extremely high diagnostic accuracy, capable of precisely identifying fault types and locations. This provides a strong guarantee for rapid fault handling and the safe operation of the system.

### 4.3. Comparison of Fault Diagnosis Methods for Various CSs

To demonstrate the advantages of the method introduced in this paper, Table 2 contrasts it with existing fault diagnosis techniques for CSs. According to the table, while reference [27] necessitates the incorporation of new hardware structures into the existing topology and is limited to diagnosing significant faults in CSs, references [6,28,29] do not require any additional hardware. However, the diagnostic approach suggested in [6] requires a considerable amount of data for training a random vector functional link network, leading to an extended diagnosis duration. In contrast, references [28,29] solely concentrate on disconnection faults in CSs, thereby covering a narrow spectrum of fault types. In comparison to these methods, the fault diagnosis method for CSs presented in this paper is applicable to scenarios encompassing incipient faults, significant faults, and complete failure faults. The method offers a faster diagnosis time, necessitates no extra hardware, and demonstrates superior diagnostic capabilities.

## 5. Conclusions

Addressing the issue of CS faults in the PSFB converter of DC charging piles, this paper innovatively proposes a fault diagnosis method based on an adaptive SMO. This approach ingeniously integrates techniques such as state augmentation and non-singular coordinate transformation to design the adaptive SMO. By introducing a novel adaptive reaching law, the observer not only achieves fast convergence but also effectively suppresses high-frequency chattering, thereby enhancing the observation accuracy of the system’s reconstructed state. In practical applications, this method enables more precise diagnosis of CS faults, significantly reducing the likelihood of misdiagnosis. Furthermore, this paper designs an adaptive threshold based on norm criteria, which not only strengthens the accuracy of fault diagnosis but also enhances its robustness. By dynamically adjusting the threshold, the method can more flexibly adapt to fault detection requirements under different operating conditions, effectively mitigating misjudgments caused by environmental variations or noise interference, thus demonstrating higher reliability and stability in practical applications.

Nevertheless, there remain several unresolved issues in this paper. Future work should further explore the following directions: First, optimize the fault diagnosis algorithm to enhance diagnostic efficiency and accuracy. Second, investigate a wider range of CS faults to broaden the applicability of the diagnostic method. Third, apply the research findings to practical DC charging systems for long-term performance evaluation and validation. It is hoped that this work will provide more comprehensive and effective safeguards for the safe operation of DC charging piles.

## Figures and Tables

**Table 1:** Key parameters of the PSFB converter.

Parameter	Value	Parameter	Value
Switching frequency	100 kHz	Output Power	400 W
Output Voltage	200 V	Output Current	22 A
Resonant Inductance	40 uH	Filter Inductance	44 nH
Filter Capacitor	500 uF	Turns Ratio	2.5

**Table 2:** Comparison of different fault diagnosis methods for CS.

Methods	Topological Structure	Additional Hardware	Diagnosis Time	Complexity	Fault Types
[6]	Three-Phase Two-Level Inverter	No	≈20 ms	High	Sensor Disconnection, Stuck, and Gain Faults
[28]	Three-Phase Two-Level Inverter	No	6–18 ms	Medium	Sensor Disconnection Fault
[27]	Three-Phase Two-Level Inverter	Yes	<12.3 ms	Medium	Sensor Stuck Fault
[29]	Single-Phase Two-Level Rectifier	No	>6 ms	High	CS Disconnection Fault
This method	PSFB Converter	No	<5 ms	Medium	CS Disconnection, Stuck, and Offset Faults

## Data Availability

Data sharing is not applicable to this article.
